# Adsorption of Heavy Metal Ions on Alginate-Based Magnetic Nanocomposite Adsorbent Beads

**DOI:** 10.3390/ma17091942

**Published:** 2024-04-23

**Authors:** Eleonora Russo, Paolo Sgarbossa, Simone Gelosa, Sabrina Copelli, Elisabetta Sieni, Marco Barozzi

**Affiliations:** 1 Department of Industrial Engineering, University of Padova, Via F. Marzolo 9, 35131 Padova, Italy; eleonora.russo.2@phd.unipd.it (E.R.); paolo.sgarbossa@unipd.it (P.S.); 2 Department of Chemistry Materials and Chemical Engineering, Politecnico of Milan, Via Luigi Mancinelli 7, 20131 Milan, Italy; simone.gelosa@polimi.it; 3 Department of Science and High Technology, University of Insubria, Via Valleggio 9, 22100 Como, Italy; sabrina.copelli@uninsubria.it; 4 Department of Theoretical and Applied Sciences, University of Insubria, Via Jean Henry Dunant 3, 21100 Varese, Italy

**Keywords:** wastewater, adsorption, nanoadsorbents, graphene oxide, magnetic nanoparticles

## Abstract

Graphene oxide and its magnetic nanoparticle-based composites are a well-known tool to remove heavy metals from wastewater. Unfortunately, one of the major issues in handling such small particles consists of their difficult removal from treated wastewater (even when their magnetic properties are exploited), due to their very small diameter. One possible way to overcome this problem is to embed them in a macroscopic biopolymer matrix, such as alginate or chitosan beads. In this way, the adsorbent becomes easier to handle and can be used to build, for example, a packed column, as in a traditional industrial adsorber. In this work, the removal performances of two different embedded magnetic nanocomposite adsorbents (MNAs) are discussed. The first type of MNA is based on ferrite magnetic nanoparticles (MNPs) generated by coprecipitation using iron(II/III) salts and ammonium hydroxide, while the second is based on a 2D material composed of MNP-decorated graphene oxide. Both MNAs were embedded in cross-linked alginate beads and used to treat artificial water contaminated with chromium(III), nickel(II), and copper(II) in different concentrations. The yield of removal and differences between MNAs and non-embedded magnetic nanomaterials are also discussed. From the results, it was found that the time to reach the adsorption equilibrium is higher when compared to that of the nanomaterials only, due to the lower surface/volume ratio of the beads, but the adsorption capacity is higher, due to the additional interaction with alginate.

## 1. Introduction

Water pollution is among the most serious threats to human life and the environment. Among the different contributors to the generation of contaminated water, industry is one of the most prominent sources. Chemical, pharmaceutical, and textile industries generate important amounts of wastewater [1], which should be properly treated to both promote the recycling of the same water and send it back to the environment with the lowest possible pollutant concentration. Wastewater can present many different species of pollutants: organic and inorganic chemicals, dusts, solids, and biological agents [2]. The most common methods for wastewater treatment include ultrafiltration, flocculation, coagulation, adsorption, and biological processes [3]. When dealing with heavy metals, typical pollutants in the textile industry related to the use of dies, it is possible to exploit chemical methods [4], but they come at high costs. Physical methods, such as adsorption, have risen in interest in the latter years, due to the possibility of making inexpensive adsorbents from wastes or low-cost reagents. Nanosized materials have found a flourishing background for adsorption and catalysis applications [5,6,7]. Nanomaterials are defined by having at least one dimension smaller than 100 nm and exhibit peculiar properties produced by the high surface area/volume ratio. In fact, if compared to massive material, the amount of surface atoms and their properties make them an optimal solution as adsorbents. As an example, Azam et al. [8] developed an adsorbent from raw date pits and chemically treated date pits to remove cadmium and chromium from wastewater. In another work, acid-modified kaolinite was used to test the removal of chromium and iron. Even Magnetic Nanoparticles (MNs) can be used as adsorbents for a wide set of metal ions [9]. Graphene oxide (GO) has also found many applications, due to its high surface area and high number of active sites [4,10]. Finally, biosorbents find many applications in this field, such as genetically engineered Saccharomyces Cerevisiae [11]. The introduction of these studies launched the implementation of historical adsorption models [12], traditionally developed to study the adsorption of gases on solid substrates, with batch experiments [13]. The idea behind this type of experiment consists of treating a sample of water at a fixed starting concentration with a fixed amount of nanoadsorbent. After a sufficient amount of time, the adsorbent is separated, and the treated water is analyzed.

Unfortunately, most of these solutions, despite exhibiting promising adsorption capacity, do not easily find application in an industrial framework. The main reason is related to the eventual regeneration process that would be required in a real wastewater treatment plant. The small dimension of nanosized adsorbents represents both an advantage and a drawback: while a high superficial area is definitely a desired feature, very small particles are also hard to remove in the treated wastewater sample. This aspect can be partially solved with the development of magnetic nanoadsorbents, which can use the imposition of a magnetic field to remove the adsorbent from clean water [9,14,15]. Another solution consists of embedding the small particles in greater structures, which can be easily segregated from the aqueous phase inside an industrial device, such as a packed column. One of the most used techniques is the generation of structures using gelation of alginate or chitosan [14,16,17]. These natural polymers are a clean and sustainable solution to develop a functionalized nanoadsorbent that could be implemented in a packed adsorber column.

In a former work of the same authors [15], a relatively cheap and simple method to develop magnetic nanoadsorbents made of nanosheets of graphene oxide with magnetic nanoparticles was studied and proposed. While proving effective to remove heavy metal solutions, such systems find little application in an industrial contest, due to their very small dimensions and difficulties in being removed from the treated solution.

In this work, previously developed nanomaterials were embedded in alginate beads, resulting in three magnetic nanocomposite adsorbents (MNAs), named BGO, B1, and B2, which can be easily removed from the treated solution or directly fixed in an adsorption column. Specifically, BGO comprises nanocomposite beads that contain GO decorated with magnetite (Fe_3_O_4_) nanoparticles, prepared by a simple coprecipitation method in the presence of graphene oxide. For comparison, MNAs B1 and B2 were prepared by dispersing the sole magnetite nanoparticles in alginate beads at different Ca^2+^ concentrations in the cross-linking solution. This study involves the application of these three adsorbents on Cr^3+^, Ni^2+^, and Cu^2+^ under different concentrations at 25 °C.

The final aim of this work was to characterize the removal performance of BGO, B1, and B2 at low contact time, which is desirable for real industrial applications, without referring to the classical adsorption isotherms (difficult to achieve because of a series of experimental concerns regarding bead handling in their hydrated form).

## 2. Materials and Methods

All reagents and solvents were purchased from Sigma-Aldrich, unless otherwise stated, and used without further purification. The magnetite nanoparticles and MNP-decorated GO nanosheets were prepared according to the procedures reported elsewhere [15].

### 2.1. Alginate Bead Preparation

**Figure 1:**
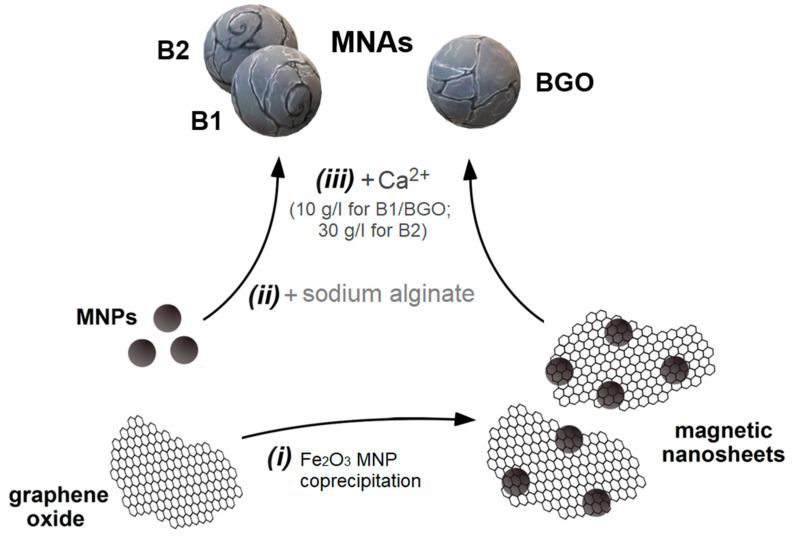
Preparation of MNAs: (i) preparation of GO nanosheets decorated with magnetic nanoparticles by coprecipitation; (ii) mixing of magnetic materials (MNP or magnetic GO) with dissolved sodium alginate; (iii) formation of the beads by cross-linking with a Ca^2+^ solution.

The general MNA preparation scheme is presented in Figure 1. Depending on the type of beads, two main pathways are available: embedded MNPs (leading to the production of B1 and B2) or embedded magnetic nanosheets (leading to the production of BGO).

Embedded MNPs B1 and B2 are prepared as follows:

(1) Dispersion of the pristine magnetite MNPs (5.0 mg/mL) in a sodium alginate (C_6_H_9_NaO_7,_ Sigma-Aldrich Italy, Milan, Italy) solution prepared by dissolving the compound in deionized water at a concentration of 10.0 g/L.
(2) Drop-wise adding of the previously prepared suspension in a cross-linking solution, obtained by dissolving either 10.0 g/L (for B1) or 30.0 g/L (for B2) of calcium chloride (CaCl_2_, Sigma-Aldrich) in deionized water. During the addition, the Ca^2+^ solution was kept under constant magnetic stirring.
(3) Mechanical recovery from the solution, washing four times with deionized water, and storage in deionized water.

Embedded magnetic nanosheets BGO are prepared as follows:

(1) Dispersion of the GO-based magnetic nanosheets (2.5 mg/mL) in a sodium alginate (C_6_H_9_NaO_7,_ Sigma-Aldrich) solution prepared by dissolving the compound in deionized water at a concentration of 10.0 g/L.
(2) Drop-wise adding of the previously prepared suspension in a cross-linking solution, obtained by dissolving 10.0 g/L of calcium chloride (CaCl_2_, Sigma-Aldrich) in deionized water. In this case, during the addition, the Ca^2+^ solution was kept under constant magnetic stirring.
(3) Mechanical recovery from the solution, washing four times with deionized water, and storage in deionized water.

All the beads were characterized with stereo microscope images (Optika SZO-4), captured with a video camera (Optika C-HP) in both wet and lyophilized form. To establish their homogeneity, shape, and mass characterization, different samples of about 30 beads were taken per type, weighted in both wet and lyophilized form. Diameter distribution was analyzed by elaborating the stereoscope images with the freeware software ImageJ (v, 1.46r), a public-domain Java image processing software.

### 2.2. Experimental Protocol

In accordance with a previous work [15], the three MNAs were tested on artificial polluted water samples prepared by dissolving the corresponding inorganic salts in deionized water. Three different heavy metal ions, commonly found in industrial effluents, were chosen:

- Cu^2+^ (Copper(II) nitrate, CuNO_3_∙3H_2_O, 99% Sigma Aldrich);
- Ni^2+^ (Nickel(II) chloride, NiCl_2_∙6H_2_O, 99% Sigma Aldrich);
- Cr^3+^ (Chromium(III) chloride, CrCl_3_∙6H_2_O, 99% Sigma Aldrich).

The starting concentration of the solution was determined by Inductively Coupled Plasma Optical Emission Spectroscopy (ICP-OES) on a Perkin Elmer Optima 4200DV (Perkin Elmer Italia S.p.A., Milano, Italy). Table 1 reports all the heavy metal starting concentrations tested.

All the prepared solutions were subsequently treated with BGO, B1, or B2 to test their adsorption removal efficiencies. The experimental protocol followed for each test (#i, i = 1:6 for all metal ions) is as follows:

(1) A flask containing 1 mL of deionized water was prepared;
(2) Then, 5 beads (B1, B2, or BGO, depending on the test) were manually added;
(3) Successively, 1 mL of the tested salt solution (#i, double concentration) was added to reach the corresponding initial concentration reported in Table 1;
(4) The flask was then mechanically shaken for 10 min at 25 °C.
  Such a time could seem very short, but it was chosen to ensure the possibility of realizing a real industrial application of the embedded nanoadsorbents (which must be used far from equilibrium conditions, characterized by poor adsorption kinetics). It was observed that such a time was sufficient to reach the “quasi equilibrium” concentration for copper for each bead type; therefore, it was used as reference contact time for all ions to make direct comparisons among the different adsorption bead performances;
(5) At the end of the experiment, 1.5 mL of solution was transferred with a micropipette into a 2 mL vial for ICP analysis. All final sample concentrations were analyzed with an ICP-OES measurement.

The pH of all the metal ion stock solutions was measured and fell in the range of 5.2–6.0.

Such a procedure was substantially the same as that one previously described in [15].

### 2.3. Adsorption Parameters for Removal Characterization

From the ICP-OES concentration data, the different adsorption performances were analyzed.

According to the literature, the adsorption data have been almost always analyzed referring to the concept of the Langmuir isotherm model. This model was historically developed to describe the gas adsorption on a solid phase adsorbate as activated carbon [18]. According to the Langmuir theory, the adsorption process is a combination of adsorption and desorption on the solid surface, promoted by the collision of the molecules in the gas phase on the available active sites. This process continues up to the equilibrium status, when adsorbate accumulation on the surface becomes equal to zero. Traditionally, the Langmuir isotherm has been associated with monolayer adsorption kinetics, whereas different models, despite being based on the same principles, describe more complex interactions, such as multi-layer and ion exchange adsorptions [13]. Due to the similarities between the gas–solid adsorption and nanoparticle–aqueous pollutant phenomena, these concepts find applications in many studies of the nanoadsorbents used to treat wastewater.

Typically, equilibrium data for an isotherm are found by using a continuous flow of fluid with a constant inlet concentration of a substance on a fixed adsorbent bed. The characterization of the equilibrium is detected by analyzing the outlet concentration. Under these conditions (and hypothesizing an ideal zero-length of the mass transfer zone), the equilibrium is reached when the outlet concentration of the pollutant becomes equal to that of the inlet.

When dealing with wastewaters, nanoadsorbents are often hard to separate due to their small dimensions, and batch experiments are preferred. In a batch experiment, a solution with a known starting concentration of pollutant is put in contact with the adsorbent, and sufficient time should be left to reach the equilibrium. After the experiment, the solid phase is separated, and the treated water is analyzed. It is worth noticing that, despite being very similar, the 2 processes (continuous and batch) show some significant differences, namely the following:

1. In a batch experiment, the starting concentration is not maintained; hence, the concentration gradient between the two phases is lower compared to the continuous one.
2. In a batch experiment, the equilibrium is reached between the adsorbent and the final concentration, while in a continuous experiment the equilibrium is between the adsorbent and the initial concentration.

Nevertheless, it is possible to extend the traditional Langmuir isotherm model reported in Equation (1) [6,15]:

(1) $$\eta_{eq}=\frac{Q_{max}\cdot b\cdot c_{e}}{1+b\cdot c_{e}}$$

where $Q_{max}$ is the maximum adsorption capacity, b is a constant, $c_{e}$ represents the equilibrium concentration of the pollutant in liquid phase, and $\eta_{eq}$ is the equilibrium load of the compound, which is adsorbed onto the MNPs, in batch experiments.

To apply the Langmuir model for a batch experiment, it is possible to modify Equation (1) as follows:

(2) $$\eta_{end}=\frac{Q_{max}\cdot b\cdot c_{end}}{1+b\cdot c_{end}}$$

where $Q_{max}$ and b are constants (resulting from a fitting/regression of experimental data), $c_{end}$ represents the final concentration of metal ion(s) (µmol/L) in the analyzed solution, and $\eta_{end}$ is the final load of metal ion(s) that is adsorbed onto the beads (µmol/mg). Assuming that the equilibrium was reached, Equation (2) then becomes

(3) $$\eta_{end}=\eta_{eq}=\frac{Q_{max}\cdot b\cdot c_{end}}{1+b\cdot c_{end}}$$

In the present work, only the final load concept will be used, linking it to the experimental ion concentrations measured according to Equation (4).

(4) $$\eta_{end}\left[\frac{\mu mol}{mg}\right]=\frac{(c_{0}-c_{end})\cdot V_{sol}}{m_{bead}}$$

where $V_{sol}$ is the volume of the batch (L), and $m_{bead}$ is the total mass of beads loaded into the batch (mg). The beads were always used “wet” or, better, in their “hydrated” form, to avoid changes in their adsorption properties. Of course, a certain amount of water, not univocally evaluable, was trapped inside the alginate matrix, altering the removal efficiency of the matrix. This is the main reason the use of Equation (4) to define the equilibrium load is not considered, by the authors of the present work, reliable for adsorption isotherm determination.

Another useful parameter for evaluating the adsorption properties of the different beads is the yield of removal (or removal efficiency), χ, which is a representation of how much of a pollutant is removed from the starting sample. The definition is straightforward and is described by Equation (5).

(5) $$\chi =\frac{c_{0}-c_{end}}{c_{0}}$$

where χ is the removal efficiency [–], $c_{0}$ is the starting concentration [µmol/L], and $c_{end}$ is the final concentration.

Note that, according to this definition alone, it is not possible to split the eventual contribution of different components of the beads (MNA, GO, alginate), and they are considered as a pseudo-mono-adsorbent.

### 2.4. Metal Ion Speciation in Water

**Figure 2:**
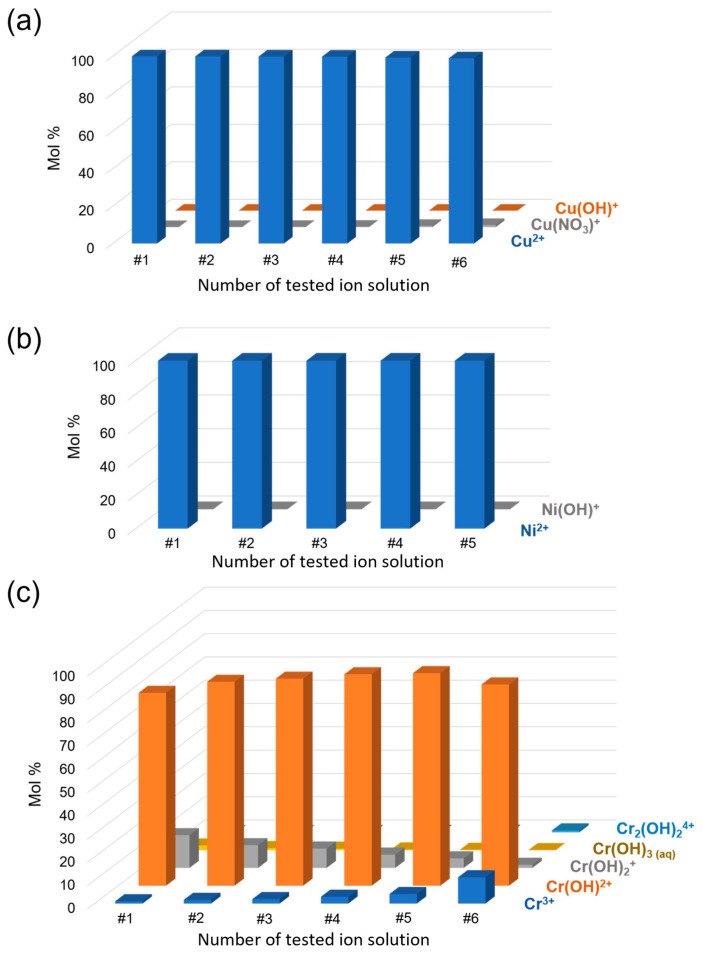
Molar distribution of copper(II) (**a**), nickel(II) (**b**), and chromium(III) (**c**) species in solution at 25 °C as determined by Visual MINTEQ Version 4.0. The x-axis refers to the solution number in Table 1.

To ascertain the nature of the metal ion species in solution at the tested concentration, a speciation analysis was carried out with the freeware software Visual MINTEQ Version 4.0 (developed by J.P. Gustafsson). The percent molar distribution of copper(II) (Figure 2a), nickel(II) (Figure 2b), and chromium(III) (Figure 2c) species in solution was evaluated at their specific pH at 25 °C.

As confirmed by the results, under test conditions, the whole amount of metal ions is in solution, with no aggregation or precipitation. If in the case of Ni(II) and Cu(II) the M^2+^ cation is the predominant species in solution, for Cr(III) the metal is more distributed in various hydroxo species, with the bis cationic [Cr(OH)]^2+^ being the most relevant.

## 3. Results

### 3.1. Bead Preparation and Characterization

The three samples of magnetic nanocomposite beads, BGO, B1, and B2, were prepared by mixing previously prepared magnetic nanomaterials and sodium alginate. BGO was prepared using a GO nanosheet decorated with magnetic nanoparticles obtained by coprecipitation, while B1 and B2 were prepared with only MNPs and alginate, by increasing the concentration of Ca^2+^ during the cross-linking step of B2 (see Section 2.1).

Table 2 shows the average composition of the different beads used. Since the beads need to remain wet to avoid irreversible drying and shrinking, their composition was determined as a mean of the alginate and nanoparticle masses used in the preparation process divided by the number of beads generated.

The same beads were used to treat samples of lab-made polluted water, prepared by dissolving heavy metal salts (copper(II) nitrate, nickel(II) chloride, and chromium(III) chloride) in deionized water.

**Figure 3:**
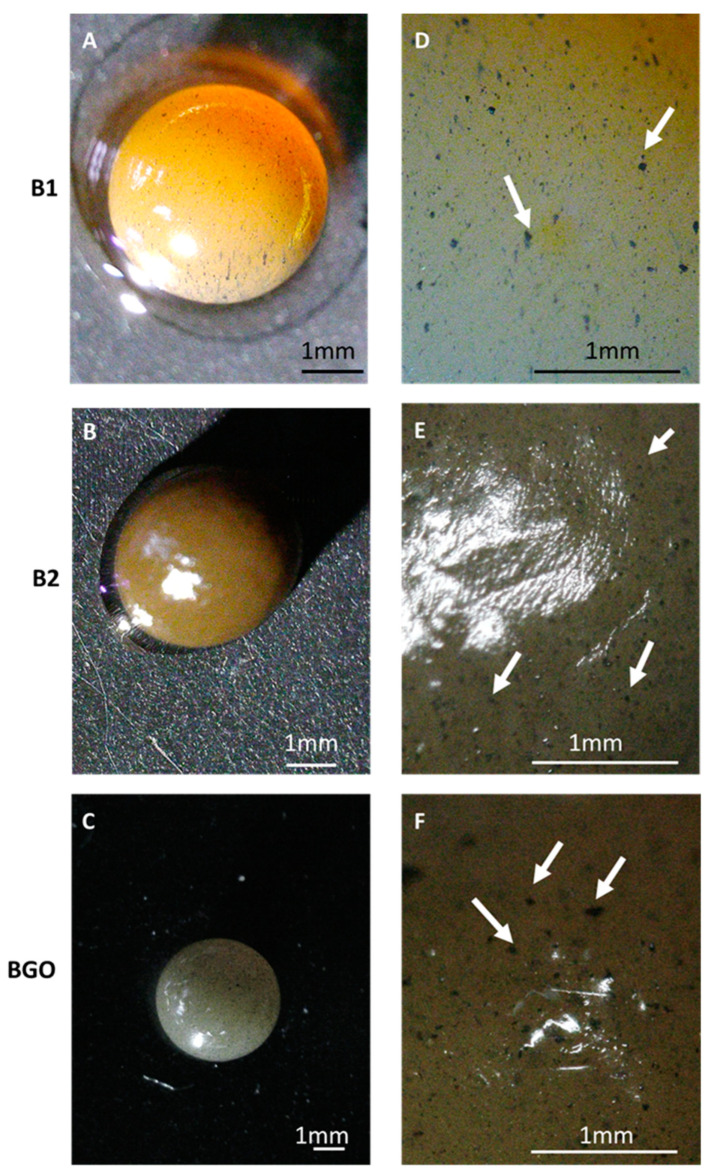
Stereoscope images of the wet beads: (**A**) B1, 2×; (**B**) B2, 1×; (**C**) BGO, 0.67×; (**D**) B1, 4.5×; (**E**) B2, 4.5×; (**F**) BGO, 4.5×. The bar indicates a length of 1 mm.

The beads were characterized in terms of morphology at the stereomicroscope. Figure 3 shows the stereoscope images of the hydrated beads, using a dark background, evidencing their regular spherical shape and smooth surface. In the magnified pictures (Figure 3D–F, some are indicated by the white arrows), the magnetic nanomaterial is clearly visible within the bead, as the black spots, with homogeneous distribution. Since the size of the spots is considerably bigger than that of the nanomaterial’s single particles [14], some aggregation within the alginate matrix during cross-linking occurred. Table 3 reports the weight of random samples of beads taken to show the reliability of the composition based on the mass balance. As can be seen from the table, the estimated average weights are comparable with the experimental results (considering the standard deviation). When comparing wet and lyophilized beads, the former have water as 97.0–97.5% of their mass.

Wet bead diameters were also estimated using ImageJ software on stereoscope pictures. B1 results in an average diameter of 3.63 mm, with a standard deviation of 0.18 mm (5.0%); B2 shows an average diameter of 3.44 mm, with a standard deviation of 0.17 mm (4.9%); and B3 results in an average diameter of 3.40 mm, with a standard deviation of 0.25 mm (7.3%). Thus, even their average density is similar: larger beads show an increased weight.

**Figure 4:**
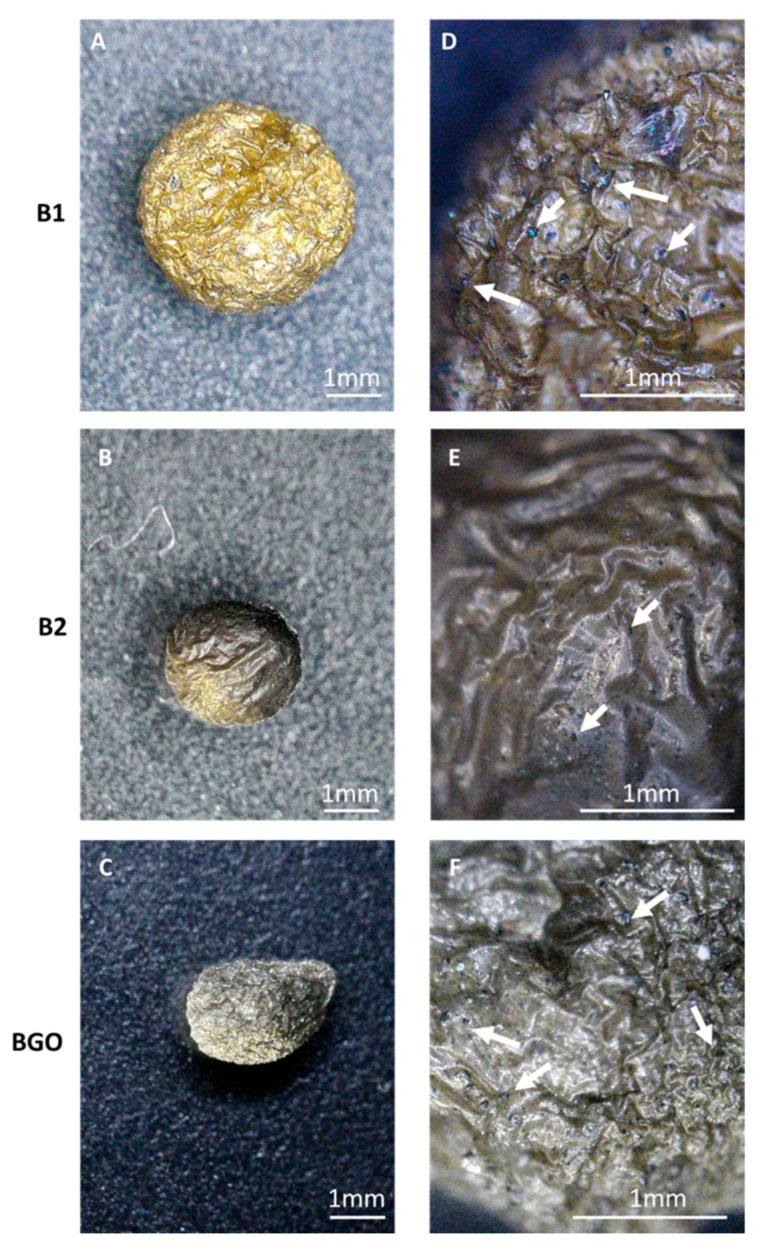
Stereoscope pictures of lyophilized beads: (**A**) B1, 1×; (**B**) B2, 1×; (**C**) BGO 1×; (**D**) B1, 4.5×; (**E**) B2, 4.5×; (**F**) BGO, 4.5×. The bar indicates a length of 1 mm.

Beads were also lyophilized for 24 h, and stereoscope pictures of the dried beads are shown in Figure 4 (white arrows indicate some of the magnetic nanoparticles close to the beads’ surface). The diameter of the beads was considerably reduced due to shrinking (from 3.4–3.6 mm to ≈2 mm). While B1 and B2 maintained a spherical shape, BGO collapsed during drying, as can be seen in Figure 4C, suggesting a less compact internal structure. In all pictures, the surface of the bead appears corrugated but still compact. The use of a different Ca^2+^ concentration in the cross-linking solution did not have a discernible effect on the morphology of the beads, as observed by comparing B1 and B2.

**Figure 5:**
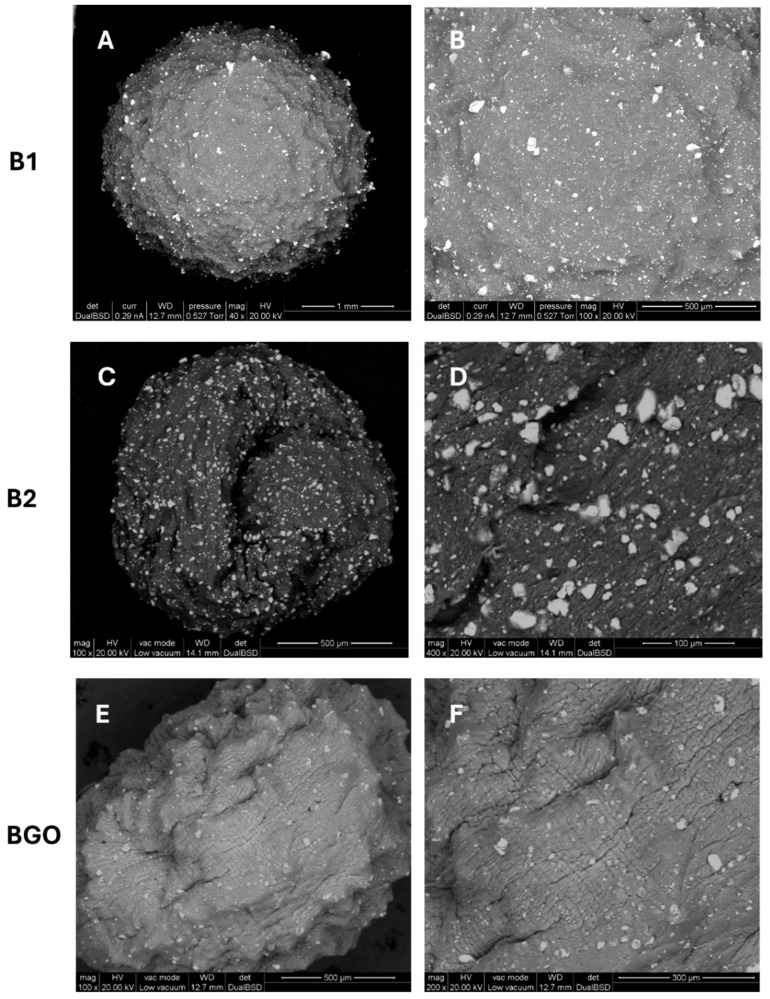
ESEM characterization of beads: (**A**,**B**) B1, (**C**,**D**) B2, (**E**,**F**) BGO.

ESEM (Environmental Scanning Electron Microscopy) analyses of the three typologies of beads were performed using a FEI-QUANTA 200 variable pressure-environmental instrument (FEI Company, Hillsboro, OR, USA), equipped with backscattered electron (BSE) detector. Despite ESEM being a technique that allows for the presence of water vapor in the sample chamber [19], we observed a rapid evaporation of the water phase in the beads with a contraction of their structure. Nevertheless, the ESEM images collected show a homogeneous distribution of the magnetic material (lighter spots in Figure 5) also on the surface of the beads. The bead B1 (Figure 5A,B) has a more regular surface and shape, while bead B2 (Figure 5C,D), obtained by using a higher (3×) concentration of Ca^2+^ in the cross-linking solution, displays cracks and inward folding of the surface.

The oblong shape (as seen in the lyophilized sample of the same type) and rough surface of BGO is probably due to the presence of the 2D graphene oxide sheet structure, which can interfere with the organization of the polymer fibers and their ionic interactions.

The elemental analysis performed by Energy dispersive X-Ray analysis (EDX, model EDAX Element-C2B) on the surface of the three samples analyzed is reported in Table 4.

The surface composition of B1 and B2 is similar and coherent with the material ratio used during their preparation, considering the presence of some residual water in their structure. On the other hand, BGO shows a much lower concentration of iron, which is in line with the lower amount of magnetite used, but also has a relatively high oxygen content, suggesting a higher hydration level of the bead compared to the previous ones. This could be related to the presence of GO, which can interact strongly with the water molecules through hydrogen bonding with its oxygenated functional groups [20].

### 3.2. Adsorption Tests: Yield and Load Analysis

The adsorption properties of the nanostructured adsorbent beads were analyzed as follows. Each test was conducted at 25 °C with the same volume of metal polluted solution (2 mL), number of beads (5), and contact time (10 min), as reported in Section 2.2.

Table 5, Table 6 and Table 7 show the tested initial solution concentrations for, respectively, copper(II), nickel(II), and chromium(III), and the corresponding final solution concentrations after the treatment with B1, B2, and BGO beads.

**Figure 6:**
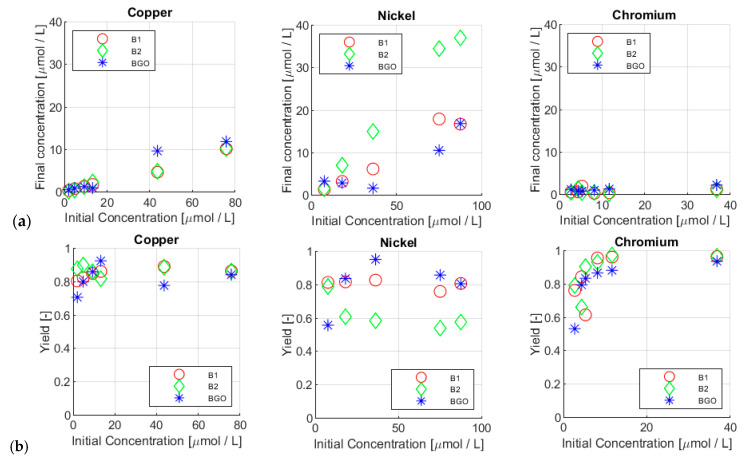
Final concentration (**a**) and yield of removal (**b**) vs. initial concentration of copper, nickel, and chromium.

In Figure 6a, the comparison between the starting and final concentrations of copper(II), nickel(II), and chromium(III) are reported for the three types of beads (red circle for B1, green diamond for B2, and blue asterisk for BGO). By looking at the data, it is possible to see that for copper and nickel the final concentration generally increases with the increase of the initial concentration, while for chromium, a general trend was not observed. This aspect will be discussed later, but the justification relies on both the adsorption kinetics and the chromium speciation: during 10 min of testing time, the adsorption of chromium does not reach equilibrium, considering the presence in solution of different hydroxo forms that may need coordination exchange processes to adsorb on the beads.

For the copper (see Table 5), the results were very similar among the different beads used (see also Figure 6a). At higher concentrations, above 2700 µg/L, BGO exhibited higher final concentrations with respect to both B1 and B2. This could be related to the lower size and surface area, as BGO was generally smaller compared to the other beads.

In the nickel(II) adsorption tests (see Table 6 and Figure 6a), the results were quite different when comparing all the adsorbents used. Particularly, the highest final concentrations are found for all the solutions treated with B2, while B1 and BGO performances seem to be quite similar.

For chromium(III) (see Table 7 and Figure 6a), the results showed that all the adsorbents were quite effective, particularly at high initial solution concentrations; in fact, very low final concentrations were always observed.

The comparison between starting and final concentration (showed in Figure 6a) is not particularly useful, as the adsorption is highly dependent on the amount of adsorbent used (about 1.6–1.85 mg_bead_/mL). For this reason, removal efficiency has to be also evaluated and discussed.

Yield of removal (or, simply, removal efficiency), χ, is a useful parameter to test how much of pollutant was removed from the starting sample.

A different affinity of a metal ion with an adsorbent is a well-known fact, represented in many studies on the subject [6,15,17].

Figure 6b reports the yields calculated from the final concentrations, for each ion and concentration tested. In this way, it was clearer to observe the behavior of the adsorbent under different conditions.

According to these data, each ion behaved differently: copper showed a very similar affinity with B1 and B2, which showed basically the same yield of removal; some differences could be traced for BGO, which was lower performing at higher concentrations. With nickel, each ion exhibited a different behavior: BGO seemed to be the highest performing bead type in terms of removal efficiency, followed by B1 and B2.

In the case of chromium, all the beads seemed to exhibit the same removal efficiency at different concentrations.

It is also worth noting that, for low concentrations, the yield tended to increase with the starting concentration for most of the samples. The maximum yields were reached for chromium, with values up to 98% with an initial concentration equal to 605 µg/L (11.6 µmol/L). This fact could indicate that the adsorption equilibrium was not yet achieved. The geometry of the beads, which was bigger compared to the functionalized nanoparticles, slowed down the adsorption process, introducing a sort of “icrokinetic bottleneck”.

**Figure 7:**
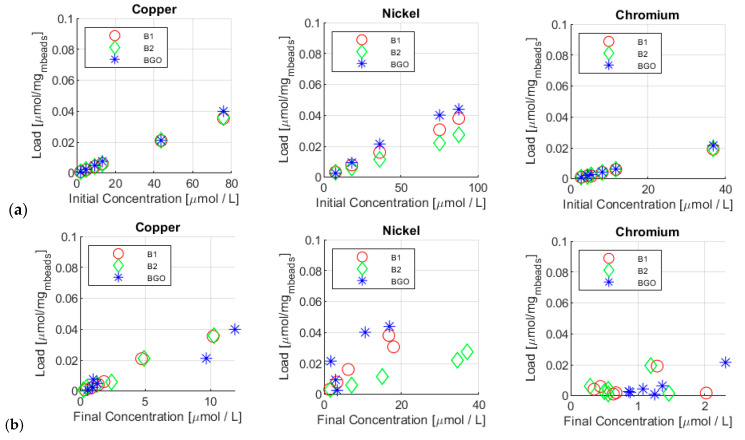
Bead load vs. initial (**a**) and final (**b**) concentration of the metal ions.

Non-equilibrium operating conditions can be also confirmed by the trend of final load of the metal on the corresponding adsorbent, $\eta_{end}$, with respect to both initial and final solution concentrations, as reported in Figure 7.

In Figure 7a, final loads are reported as a function of the initial concentration, while, in Figure 7b they are reported as a function of the final concentration.

In Figure 7a, it is possible to identify that, for each test carried out, the load increased with the initial concentration. No plateau region can be observed. This is additional evidence that the equilibrium state was not reached in such a short time. However, it is reasonable to suppose that the equilibrium state was not far, at least for copper, as the load increased with the increase of the initial concentration. This aspect is of crucial importance because, for real industrial adsorbers, saturation time is a crucial parameter.

Figure 7b shows instead the load vs. the final concentration of ions that should be, in theory, the equilibrium concentration. From the results, it was highlighted that not only was the equilibrium state not yet achieved (with an exception for copper), but the bead saturation was also not yet achieved, as the loads increased with the final concentration but they did not reach the typical plateau, indicating total active site saturation. Comparing these results with other studies, some interesting aspects can be observed. In a similar paper [17], an adsorbent made of sawdust chitosan nanocomposite beads was applied to treat copper and nickel aqueous solutions. In this work, the adsorbent concentration was equal to 20 mg/mL (1 g on 50 mL of solution) [17], with a tested concentration in the range of 20–200 mg/L. In the current work, a bead concentration in the range of 1.6–1.8 mg/mL was used, along with an ion concentration in the range of 0.1–5 mg/L. Hence, the adsorbent/pollutant ratios are comparable. According to this study, equilibrium was reached in 70 min. Compared to our study, even after 10 min of stirring, the yields of removal were 50% higher for each ion studied.

### 3.3. Comparison with Nanosheet and MNPs without Alginate

**Figure 8:**
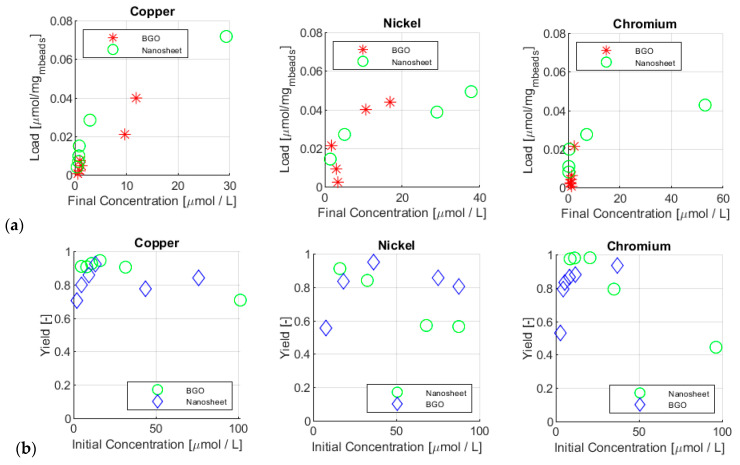
Comparison of the removal loads (**a**) and yields (**b**) between magnetic GO nanosheets and BGO for copper, nickel, and chromium.

In a former work of the same authors [15], a set of pure MNPs functionalized with a GO nanosheet was tested under conditions similar to the ones of this work. The results of this former work basically represented a trial of the same nanoparticles embedded inside the alginate beads. The adsorbent closest to the nanosheet used was BGO, and comparison between it and the nanosheet alone was provided in this analysis. Figure 8a shows the ion loads vs. initial concentration. From these graphs, it is particularly clear that neither site saturation nor equilibrium was reached with the beads. With the nanosheet, where the equilibrium was reached, the loads reached the plateau, typical of saturation, and they constantly increased with the final concentration. With BGO, this did not occur. This was a positive aspect, as it highlighted the potential of adsorption of the beads, which acted for 10 min only.

Figure 8b shows instead the removal capacities. In this case, it was interesting to highlight that, for BGO, the yield kept increasing in almost every test, highlighting the fact that the results were far from saturation. However, it is important to remember that each load was estimated by using the whole mass of beads, which was composed of only 15–30% of MNPs. BGO was the sample with the lower amount of MNPs, at only 14.6% in mass. Since loads and yields were of the same order of magnitude, this meant that MNPs were not the only participant in the adsorption process.

**Figure 9:**
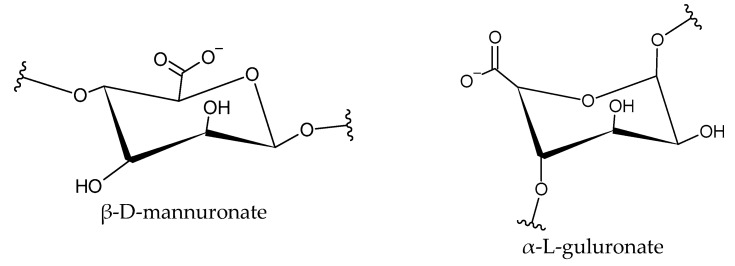
Monomers of alginate.

This is a crucial aspect to be further investigated. Alginate is a linear anionic polysaccharide chain composed of a 1,4-glycosidic bond linked α-L-guluronic acid residues and β-D-mannuronic acid residues (Figure 9), in different proportions and arrangements [16].

**Figure 10:**
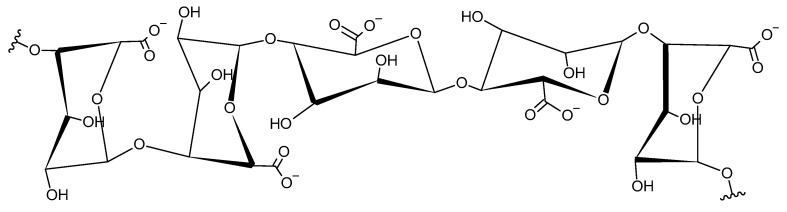
Example of a structure of alginate.

Alginate can develop different structures, composed by different sequences of β-D-mannuronate and L-guluronate, such as the example represented in Figure 10.

When used to generate beads, alginate is cross-linked with the use of a hardener solution, typically CaCl_2_. The resulting structure maintains carboxylate and hydroxy groups, representing potential active sites for adsorption [14]. This means that it is actually improper to model an adsorption isotherm for a bead with a single model, since multiple, different active sites are available. In order to provide a proper model, whole sets of experimental data with alginate only, MNPs only, and beads need to be implemented and studied, proposing the best model for each component, as well as a mixed model, such as a bi-Langmuir model [21].

## 4. Conclusions

In this work, a set of magnetic nanocomposite adsorbents based on magnetic nanomaterials (magnetite nanoparticles and graphene oxide decorated with them) embedded in alginate beads were tested at low contact time (10 min) at 25 °C. The results, even if preliminary, showed a good potential of the nanoadsorbents proposed (B1 seems to be the best-performing bead considering all the metal ions tested). At first, the adsorption capacity was potentially high, but it required proper modeling for industrial application; alginate competed in the adsorption process, and it must be taken in account. Also, it was evident that 10 min was not a sufficiently high contact time to reach the equilibrium state, necessary to evaluate the adsorption isotherms. However, based on the comparison with similar works, the time to reach equilibrium should not be significantly higher, providing a good compromise for an industrial adsorber. Finally, such an adsorbent could provide a powerful tool to treat wastewater. The beads were very homogeneous in shape and size, easy to reproduce, and cost-effective. They could be easily implemented in a packed column, which is a well-known technology for industrial adsorbers. With their magnetic behavior, they could be also magnetically stirred or heated by induction (to improve the regeneration of the adsorbent). However, before reaching such an application, it will be important to study in detail the equilibrium, developing the most suitable isotherm model to describe the behavior of the beads and optimizing the working conditions.

## Figures and Tables

**Table 1:** Target and analytical concentrations of the heavy metal ions in the tested solutions (Th: theoretical, Eff: effective).

Sample	Th. Conc. Cu [µg/L]	Eff. Conc. Cu [µg/L]	Th. Conc. Ni [µg/L]	Eff. Conc. Ni [µg/L]	Th. Conc. Cr [µg/L]	Eff. Conc. Cr [µg/L]
#1	100	123 ± 1	400	443 ± 1	100	139 ± 1
#2	300	302 ± 1	1000	1060 ± 10	200	225 ± 1
#3	600	587 ± 1	2000	2120 ± 10	300	273 ± 1
#4	800	838 ± 1	4000	4394 ± 10	400	423 ± 1
#5	3000	2770 ± 10	5000	5123 ± 10	600	605 ± 1
#6	5000	4820 ± 10	-	-	2000	1915 ± 10

**Table 2:** Average composition of the beads tested.

#	B1	B2	BGO
Composition	MNP + Alginate	MNP + Alginate	MNP + Alginate + GO
Weight	0.74 mg/bead	0.75 mg/bead	0.64 mg/bead
MNP	0.22 mg/bead	0.22 mg/bead	0.10 mg/bead
Alginate	0.52 mg/bead	0.53 mg/bead	0.53 mg/bead
GO	-	-	0.010 mg/bead

**Table 3:** Average size in dimension and weight of the beads tested.

#	B1		B2		BGO	
	Wet	Dry	Wet	Dry	Wet	Dry
# of beads	27	27	29	29	34	34
Average Weight	23.4 mg	0.7 mg	23.1 mg	0.7 mg	22.6 mg	0.6 mg
Std. Dev.	2.5 mg	0.1 mg	1.6 mg	0.1 mg	3.6 mg	0.1 mg
Std. Dev. (%)	10.5%	12.4%	6.8%	13.1%	16.0%	13.4%

**Table 4:** EDX elemental analysis.

Element	Weight %—B1	Weight %—B2	Weight %—BGO
C	22.08	26.06	12.77
O	61.65	55.57	83.21
Ca	5.87	5.62	2.03
Fe	10.40	12.76	1.99

**Table 5:** Results for adsorption with Cu(II) polluted samples; the names of the samples are in bold.

	#1	#2	#3	#4	#5	#6
Initial Cu Conc.	123 ± 1 µg/L 1.94 µmol/L	302 ± 1 µg/L 4.75 µmol	587 ± 1 µg/L 9.24 µmol/L	838 ± 1 µg/L 13.2 µmol/L	2770 ± 10 µg/L 43.6 µmol/L	4820 ± 10 µg/L 75.8 µmol/L
Bead type	**B1**	**B1**	**B1**	**B1**	**B1**	**B1**
Final Cu Conc.	24 ± 1 µg/L 0.377 µmol/L	53 ± 1 µg/L 0.833 µmol/L	88 ± 1 µg/L 1.38 µmol/L	115 ± 1 µg/L 1.81 µmol/L	299 ± 1 µg/L 4.70 µmol/L	648 ± 1 µg/L 10.2 µmol/L
Bead type	**B2**	**B2**	**B2**	**B2**	**B2**	**B2**
Final Cu Conc.	15 ± 1 µg/L 0.236 µmol/L	30 ± 1 µg/L 0.472 µmol/L	82 ± 1 µg/L 1.29 µmol/L	152 ± 1 µg/L 2.39 µmol/L	311 ± 1 µg/L 4.89 µmol/L	652 ± 1 µg/L 10.2 µmol/L
Bead type	**BGO**	**BGO**	**BGO**	**BGO**	**BGO**	**BGO**
Final Cu Conc.	36 ± 1 µg/L 0.566 µmol/L	60 ± 1 µg/L 0.94 µmol/L	82 ± 1 µg/L 1.29 µmol/L	62 ± 1 µg/L 0.976 µmol/L	615 ± 1 µg/L 9.68 µmol/L	755 ± 1 µg/L 11.9 µmol/L

**Table 6:** Results for adsorption with Ni(II) polluted samples; the names of the samples are in bold.

	#1	#2	#3	#4	#5
Initial Ni Conc.	443 ± 1µg/L 7.55 µmol/L	1060 ± 1 µg/L 18.1 µmol/L	2120 ± 1 µg/L 36.1 µmol/L	4394 ± 10 µg/L 74.9 µmol/L	5123 ± 10 µg/L 87.3 µmol/L
Bead type	**B1**	**B1**	**B1**	**B1**	**B1**
Final Ni Conc.	82 ± 1 µg/L 1.40 µmol/L	193 ± 1 µg/L 3.29 µmol/L	364 ± 1 µg/L 6.20 µmol/L	1053 ± 1 µg/L 17.9 µmol/L	982 ± 1 µg/L 16.7 µmol/L
Bead type	**B2**	**B2**	**B2**	**B2**	**B2**
Final Ni Conc.	93 ± 1 µg/L 1.58 µmol/L	415 ± 1 µg/L 7.07 µmol/L	881 ± 1 µg/L 15.0 µmol/L	2022 ± 1 µg/L 34.4 µmol/L	2171 ± 1 µg/L 37.0 µmol/L
Bead type	**BGO**	**BGO**	**BGO**	**BGO**	**BGO**
Final Ni Conc.	196 ± 1 µg/L 3.34 µmol/L	172 ± 1 µg/L 2.93 µmol/L	100 ± 1 µg/L 1.70 µmol/L	621 ± 1 µg/L 10.6 µmol/L	990 ± 1 µg/L 16.9 µmol/L

**Table 7:** Results for adsorption with Cr(III) polluted samples; the names of the samples are in bold.

	#1	#2	#3	#4	#5	#6
Initial Cr Conc.	139 ± 1 µg/L 2.67 µmol/L	225 ± 1 µg/L 4.33 µmol/L	273 ± 1 µg/L 5.25 µmol/L	423 ± 1 µg/L 8.13 µmol/L	605 ± 1 µg/L 11.6 µmol/L	1915 ± 1 µg/L 36.8 µmol/L
Bead type	**B1**	**B1**	**B1**	**B1**	**B1**	**B1**
Final Cr Conc.	33 ± 1 µg/L 0.635 µmol/L	35 ± 1 µg/L 0.673 µmol/L	105 ± 1 µg/L 2.02 µmol/L	18 ± 1 µg/L 0.346 µmol/L	23 ± 1 µg/L 0.44 µmol/L	67 ± 1 µg/L 1.29 µmol/L
Bead type	**B2**	**B2**	**B2**	**B2**	**B2**	**B2**
Final Cr Conc.	29 ± 1 µg/L 0.558 µmol/L	76 ± 1 µg/L 1.46 µmol/L	26 ± 1 µg/L 0.500 µmol/L	29 ± 1 µg/L 0.558 µmol/L	15 ± 1 µg/L 0.288 µmol/L	62 ± 1 µg/L 1.19 µmol/L
Bead type	**BGO**	**BGO**	**BGO**	**BGO**	**BGO**	**BGO**
Final Cr Conc.	65 ± 1 µg/L 1.25 µmol/L	46 ± 1 µg/L 0.885 µmol/L	45 ± 1 µg/L 0.865 µmol/L	56 ± 1 µg/L 1.08 µmol/L	71 ± 1 µg/L 1.36 µmol/L	120 ± 1 µg/L 2.31 µmol/L

## Data Availability

Data are available upon request to the corresponding authors.
